# Locomotion (behavioural) test in the terrestrial oligochaetes *Eisenia* exposed to carbamate model substance

**DOI:** 10.1007/s10646-025-02870-3

**Published:** 2025-03-15

**Authors:** Antonio Calisi, Mario Angelelli, Davide Gualandris, Davide Rotondo, Giorgio Mancinelli, Francesco Dondero

**Affiliations:** 1 Department of Science and Technological Innovation, University of Eastern Piedmont, Viale Michel 11, 15121 Alessandria, Italy; 2 Department of Social and Human Sciences, University of Salento, Complesso Studium 2000, via di Valesio, 73100 Lecce, Italy; 3 Department of Biological and Environmental Science and Technologies, University of Salento, Via prov.le Lecce-Monteroni, 73100 Lecce, Italy

**Keywords:** Earthworm motility, Behaviour, Neurotoxic effect, Bioindicator

## Abstract

This study examines earthworm behaviour by combining locomotion-based motility assessments with evaluations of acetylcholinesterase (AChE) inhibition. Motility analysis revealed significant differences in the two-dimensional movement patterns of earthworms exposed to carbamate pesticides compared to those in the control group, indicating altered trajectories. AChE assays demonstrated a pronounced inhibitory effect on enzyme activity in exposed earthworms relative to unexposed individuals. Both univariate and multivariate analyses confirmed that the pesticide contaminant significantly affects AChE activity as well as the quantitative and directional characteristics of earthworm movement. These results suggest that behavioural testing in earthworms is a valuable tool for understanding the impact of pesticides on non-target organisms and the environment.

## Introduction

Soil is defined as a natural body that supports life, resulting from complex biogeochemical and physical processes; that contains mineral particles mixed with organic substances, water, air, and microorganisms (Hartemink, 2016). This environmental compartment accumulates most of the organic and inorganic pollutants released into the biosphere (Leclerc et al. 2019).

Pollutants in soil represent a serious hazard to organisms living in both belowground and aboveground systems. Exposure and effect assessment of soil pollutants is therefore necessary for decision-making related to ecosystem protection and establishment of remediation procedures.

Soil risk assessment cannot be based solely on chemical analysis of pollutants in the soil compartment (Grassi et al. 2022) because this approach does not provide an indication of deleterious effects of contaminants on the biota. Accordingly, methods other than the chemical analysis of environmental contaminants are required for a better environmental risk assessment of soil pollution. Furthermore, the primary toxicity of a contaminant exerted at low levels of biological organization will subsequently have repercussions on subsequent levels of hierarchical organization (Dondero and Calisi 2015, Calisi et al. 2019). Thanks to rapid ecological answers, behavioural tests are considered a promising tool for ecological risk assessment and environmental toxicology (Amiard-Triquet and Amiard, 2013, Dondero and Calisi 2015, Calisi et al. 2019, Ågerstrand et al. 2020). Their use can reveal direct effects on the nervous systems or sensory organs caused by different types of pollutants or mixtures (Sanchez-Hernandez, 2011; Jouni et al. 2018). Among the different behavioural responses generally considered, changes in locomotion and movement capacity are the most widely employed for the evaluation of soil contamination (Jouni et al. 2018, Zidar et al. 2019, Ågerstrand et al. 2020).

In recent years, successful behavioural ecotoxicological tests have been carried out on earthworms (Brami et al. 2017, González-Alcaraz et al. 2019, Ågerstrand et al. 2020), indicating that the methodological approach may be particularly effective with this group of invertebrates.

*Eisenia fetida* is an earthworm species widely used for toxicity bioassays (ISO, 1993, 1996, 2008) and biomarker application in soil biomonitoring (Sanchez Hernandez, 2019, Vig et al. 2022, Alves et al. 2022, Calisi et al. 2025). Among soil pollutants, Methiocarb is a methyl carbamate insecticide and molluscicide widely used to control pests in different types of crops in typical concentrations of 112–224 mg/m^2^, and as bird repellent on seeds. Its use in agriculture has been banned since 2020 in European Union (EU) due to a significant risk for health and potential environmental impact (EFSA, 2018). Methiocarb appears to be toxic to earthworms, with an LC50 value of 1.322 g/kg (EFSA 2006, Lewis et al. 2016). Nevertheless, methiocarb is still relevant to risk assessment as a model substance acting as a reversible acetylcholinesterase inhibitor, disrupting the nervous system of target and non-target organisms. Acetylcholinesterase (AChE) plays a very important role in the function of the neuromuscular system as it prevents continuous muscle contractions (Gu et al. 2021; Zhang et al. 2019). Inhibition of AChE causes dysfunction in organisms such as behavioural and locomotory changes, paralysis and death (Lionetto et al. 2011; Rotondo et al. 2025). AChE activity has been used to analyse soil contamination effects mediated by a broad spectrum of carbamates (Sanchez-Hernandez, 2011; Lionetto et al. 2011; Dondero and Calisi, 2015; Sanchez Hernandez, 2019). Therefore, AChE is often used as a biomarker for studying toxicity (Kim et al. 2011).

The primary objective of this study was to examine earthworm behaviour concerning locomotion responses using a standardized approach. The locomotion test was developed to evaluate various motility parameters in earthworms to verify the damage caused by carbamates to the neuromuscular system, which is directly involved in locomotion and behavioural functions (Cohen and Sanders, 2014). Earthworm peristaltic and crawling movements are mediated by the parallel contraction and expansion of circular and longitudinal muscles through the modulation of neurotransmitters (Liu et al. 2023). Various locomotion variables, including path length, fractal dimension (FractalD), mean cosine, path turn in the same direction (PTurn), and correlation between adjacent turn angles (CorrT), were analysed in earthworms exposed to methiocarb. Additionally, to verify the correlated effect on the neuromuscular system, the study assessed acetylcholinesterase (AChE) activity. We employed a rigorous univariate and multivariate statistical framework to hypothesize that, although earthworms exhibit a repulsive response to the contaminant by attempting to move away from its source, the pesticide’s effects can be measured equally at both the physiological level (AChE activity) and the behavioral level (altered movement).

## Materials and methods

### Animals and treatments

A homogeneous batch of *Eisenia fetida* (*n* = 90) of similar sizes (wet weight after gut content clearance: 0.63 ± 0.06 g, mean ± SE) was acclimated for 48 h in 2 containers (45 animals per container), each partially filled with 3 kg of soil at 18 ± 1 °C and 16:8 h light/dark regime. The artificial standard soil utilized was a mixture of 10% sphagnum peat, 20% kaolin clay, and 70% sand (OECD 1984, 2004).

The soil moisture content was adjusted to 45% of the water holding capacity with deionized water. The initial pH was 5.6, subsequently adjusted to 6.0 with calcium carbonate. After this acclimatization period, organisms were exposed for 72 h to a single sub-lethal concentration (100 mg/kg a.s.) of methiocarb (EFSA 2006, EFSA, 2018; Lewis et al. 2016) according to the following experimental design: 2 conditions namely, methiocarb treated and an untreated reference control with 10 animals X 3 replicates. The remaining organisms (30) were used to determine the studied parameter (locomotion variables and acetylcholinesterase activity).

Analytical grade methiocarb (Pestanal®) was used (Sigma Aldrich®, Merck, Darmstadt, Germany). Aqueous solutions of methiocarb were prepared by dissolving the compound in 95% ethanol, followed by dilution with ultrapure water (Souiad et al. 2020). The toxicants were incorporated into the soil at the beginning of the exposure experiment in accordance with OECD test procedures (OECD, 1984, 2004).

### Motility test

The experimental setup for investigating the movement behavior of *Eisenia fetida* specimens was adapted from methods detailed for other species (Longo et al. 2016). Movement paths were recorded for specimens in both control and treatment containers at the beginning of the exposure period (time 0) and after 72 hours (time 72 h). A circular arena made of a 300 mm wide white plastic dish and 1 cm high was positioned under a VGA CCD webcam (SPC900NC- Philips, Amsterdam-Netherland) connected to a laptop located azimuthally at a distance of 50 cm, with a field-of-view of 320 mm in diameter. At the start of each trial, a single organism was placed in the middle of the plate. After acclimating the organism for 1 min, the video recording was started and terminated after 2 min. At the end of each trial, the specimen was removed, and the arena was thoroughly cleaned by washing twice with 70 and 90% ethanol, followed by flushing with distilled water and drying to eliminate chemical cues.

The locomotion variables assessed for each trial included path length, fractal dimension (FractalD), mean cosine, path turn in the same direction (PTurn), and correlation between adjacent turn angles (CorrT). Path Length represents the total length of the path travelled by the organism during a specific period of locomotion. Fractal Dimension (FractalD) is a measure of the complexity or irregularity of the organism’s movement trajectory. Fractal dimension quantifies how the detail in a pattern changes with the scale at which it is measured. Mean Cosine is a measure indicating the average alignment of the organism’s movement direction over time. It reflects the degree of straightness or linearity in the trajectory. Path Turn in the Same Direction (PTurn) instead is the frequency with which the organism makes turns in the same direction along its path. This parameter provides insight into the consistency of directional changes during locomotion. Correlation Between Adjacent Turn Angles (CorrT) is a measure of the relationship between consecutive turn angles in the organism’s movement trajectory. It assesses the degree of correlation or pattern in the turning behaviour of the organism. The procedure was repeated for all specimens. Video files for each specimen (640 × 480 pixels, MP4 format) were analyzed to generate Free Movement Paths (FMPs) using the R package trajr (v.1.3.0, McLean and Skowron Volponi, 2018).

### AChE activity measurement

Acetylcholinesterase (AChE) activity was measured in all organisms following the motility test at two time points: initially at time 0 (*n* = 30) and 72 h post-exposure (*n* = 30 for both exposed and unexposed groups). The enzyme activity was measured in tissue extracts obtained from whole organisms using the Acetylcholinesterase Reagent kit (Ikzus, Italy). Tissues, snap-frozen after exposure, were stored at −80 °C until processing. Frozen tissues were homogenized in Tris-HCl buffer (0.1 M, pH 7.5, 0.1% Triton-X100) using an Ultra-Turrax blend homogenizer in the presence of leupeptine. The resulting homogenate underwent centrifugation at 9000 g for 20 min at 4 °C. The supernatant was collected and used for AChE activity determination. AChE activity was quantified spectrophotometrically following the method described by (Elmann et al. 1961).

### Data analysis

Data were analyzed by ANOVA. The assumptions of normality and homogeneity of variances were checked using Shapiro-Wilk and Levene tests. Post-hoc comparisons were envisaged using Tukey HSD tests. For multivariate analyses, we also used MANOVA (Anderson, 2001), which is implemented in R. While the coefficient estimates associated with the 72 h control and treatment groups coincide with the estimates from individual ANOVAs, MANOVA takes into account potential correlations among the response variables, leading to more powerful tests for the overall significance of the treatment effect. Multivariate normality was checked using Henze-Zirkler test (Thode, 2002); homogeneity of variance-covariances was assessed using Box’s M test for equivalence of covariance matrices.

## Results

**Fig. 1:**
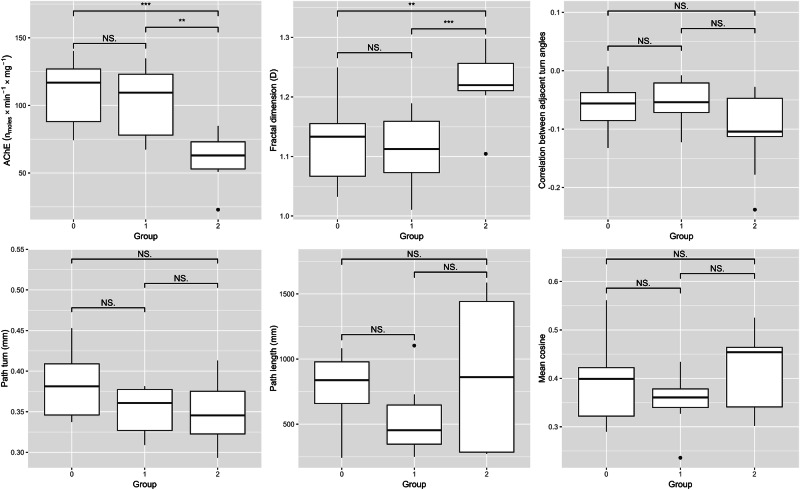
Boxplots of different measured variables (Ache, FractalD, CorrT, Pturn, Path length, mean cosine) across three groups: Group 0 (control) includes untreated earthworms at time 0, while Groups 1 and 2 consist of untreated and methiocarb-treated earthworms, respectively, after 72 h (0 = untreated, control time 0 h; 1 = untreated, control time 72 h; 2 = methiocarb treated, time 72 h). Statistical significance in terms of adjusted *p*-value (**p < 0.01; ***p < 0.001; NS not significant)

We identified significant differences between these groups using a two-way ANOVA, followed by Tukey’s post-hoc test. Indeed Fig. 1 shows that earthworms exposed to methiocarb followed altered trajectories, measured as changes in mean fractal dimension, compared to controls (*p* < 0.001). Moreover, acetylcholinesterase activity differed between control specimens and methiocarb-exposed earthworms (100 mg/kg), with a strong inhibitory effect on acetylcholinesterase activity in exposed animals after 3 days (*p* < 0.01).

In addition to identifying statistically significant treatment effects, we assessed their strength by means of univariate data analysis on each response variable. The results are summarized in Table 1, including effect sizes indicated by η^2^ and ω^2^ coefficients. These effect sizes highlight that the contaminant had substantial impact, particularly on acetylcholinesterase activity and fractal dimension, with very strong effects (η^2^ > 0.4, ω^2^ > 0.35).

**Table 1:** Univariate ANOVA statistics

Variable	$T=72h$, control		$T=72h$, treated		*F* (2, 23)	${\eta }^{2}$	${\omega }^{2}$
	*E*	*SE*	*E*	*SE*			
AChE	−8.491	11.304	−49.214^***^	10.996	11.4^***^	0.50	0.44
Fractal dimension	−0.014	0.032	0.102^*^	0.031	8.203^**^	0.42	0.36
Corr. adj. turn angles	0.003	0.025	−0.048	0.025	2.646	0.19	0.11
Path turn	−0.033	0.019	−0.034	0.018	2.211	0.16	0.09
Path length	−223.466	192.817	138.085	187.060	1.777	0.13	0.06
Mean cosine	−0.036	0.038	0.022	0.037	1.173	0.09	0.01
Mean step size (B-Ct)	−0.042	0.167	−0.023	0.162	0.033	~ 0	~ 0

Coefficient estimates, statistics, and effect sizes for individual response variables under consideration. E = coefficient estimates, SE = residual standard error. ${\rm{F}}(2,\quad 23)$ is the value of the F-statistics that determines the p-value *p* and significance of the coefficients, which is denoted by “***” ($0\,\le \,p\,\le \,0.001$), “**” ($0.001\, < \,p\,\le \,0.01$), “*” ($0.01\, < \,p\,\le \,0.05$), or “.” ($0.05\, < \,p\,\le \,0.1$). The effect sizes *η*^2^ and *ω*^2^ quantify the amount of variance in the response variables that is explained by the model. For the mean step size, the results refer to the Box-Cox transformation (B-Ct) of the original variable

The variables contributing to group differences in Table 1 were identified through pairwise comparisons for each response variable using Tukey’s HSD method, whose outcomes are summarized in Table 2. These comparisons reveal that significant differences mainly occur between unexposed (both at time 0 and after 72 h.) and methiocarb-exposed earthworms (*p* < 0.01).

**Table 2:** Pairwise comparison from each response variable in MANOVA

Variable	Compared groups		E	CI (Low)	CI (Up)	Adj. p-value
	1st group	2nd group				
AChE	0	1	−8.491	−36.800	19.817	0.736
	0	2	−49.214	−76.677	−21.750	**0.0005**
	1	2	−40.722	−69.031	−12.413	**0.0041**
Fractal dimension	0	1	−0.014	−0.093	0.066	0.901
	0	2	0.102	0.025	0.179	**0.008**
	1	2	0.116	0.036	0.195	**0.004**
Correlation between adjacent turn angles	0	1	0.003	−0.061	0.066	0.994
	0	2	−0.048	−0.110	0.013	0.145
	1	2	−0.051	−0.115	0.013	0.134
Path turn	0	1	−0.033	−0.079	0.014	0.205
	0	2	−0.034	−0.079	0.012	0.173
	1	2	−0.001	−0.048	0.046	0.999
Path length	0	1	−223.47	−706.34	259.41	0.489
	0	2	138.09	−330.37	607.55	0.744
	1	2	361.55	−121.33	844.43	0.169
Mean cosine	0	1	−0.036	−0.131	0.059	0.622
	0	2	0.022	−0.070	0.114	0.819
	1	2	0.059	−0.037	0.153	0.299

Statistically significant differences between groups are highlighted in bold. *P*-values are adjusted to take into account the multiple comparisons, and values smaller than 0.05 are highlighted in bold

*E* coefficient estimates, *CI* confident interval. 0 = 0h, 1 = $72{\rm{h}}$ control, 2 = $72{\rm{h}}$ treated

To further investigate the significance of the treatment while accounting for combinations of measured parameters, we conducted a MANOVA after applying Box-Cox transformation for the normality correction. This multivariate analysis revealed a significant group effect, yielding an overall p-value of *p* = 0.002. In addition, a second MANOVA was performed using a more parsimonious model using only the response variables that showed significant individual effects in Table 1 (acetylcholinesterase activity and fractal dimension), finding *p* = 0.008.

## Discussion

Our study reveals significant neurological and behavioural (locomotion) effects of carbamate on earthworms at sub-micromolar levels. Understanding both movement and physiological mechanisms provides sensitive and ecologically relevant data, essential for assessing the potential impacts of pollutants on ecosystem organisms. Earthworm movement is driven by external stimuli and is consequently altered under adverse conditions, such as the presence of hazards or the detection of soil pollutants, which can be detrimental or even lethal to the organisms (Calisi et al. 2019; Rotondo et al. 2025).

We investigated earthworm behaviour responses in locomotion by means of a simple approach based on a low-resolution camera and freeware video analysis software combined with multivariate and univariate statistics. The selected parameters collectively offer a comprehensive analysis of the characteristics and patterns of the organism’s locomotion, aiding in the understanding of its behaviour and responses to external stimuli or conditions such as pesticide exposure. Alteration in earthworm’s motility, observed in the field after pesticide application, was (Mather and Christensen, 1998) viewed as a consequence of avoidance. In previous studies, imidacloprid, a widely used pesticide, was employed to evaluate the effects of certain contaminants on the motility of oligochaetes (Dittbrenner et al. 2010; Cang et al. 2017). Additionally, this pesticide was utilized to investigate whether changes in motility are associated with physiological parameters such as acetylcholinesterase inhibition (Wang et al. 2015; Teng et al. 2022). In our study, results obtained from post-hoc comparisons showed that in earthworms exposed to methiocarb, there were alterations quantified as a difference in the fractal dimensions of movement trajectories. Regarding the increase in fractal dimension in earthworms, it’s essential to understand the concept of fractals in the context of movement trajectories. Fractals are geometric shapes that exhibit self-similarity at different scales (McLean and Skowron Volponi, 2018). In the context of movement trajectories, fractal dimension quantifies the degree of irregularity or complexity in the path followed by an organism (McLean and Skowron Volponi, 2018). A higher fractal dimension indicates greater complexity or irregularity in the movement trajectory (Karimui, 2021). Therefore, an increase in fractal dimension in earthworms exposed to methiocarb suggests that their movement trajectories became more irregular, or complex compared to unexposed earthworms.

This could manifest as more convoluted or erratic paths during locomotion, possibly reflecting disturbances in neurophysiological processes or behavioural responses to pesticide exposure (Prado et al. 2023; Bandeira et al. 2023; Soose et al. 2023). For example, (Soose et al. 2023) found that exposure to methiocarb caused hyperactivity and increased total distance moved in aquatic species *Gammarus pulex*. Similar to our study, (Bandeira et al. 2023) demonstrated that in collembolans, locomotion trajectories were altered in exposed organisms compared to non-exposed ones. In our research, we combined motility analysis with the assessment of acetylcholinesterase (AChE) activity inhibition. As expected, methiocarb exposure resulted in significant inhibition of AChE. Moreover, multivariate analyses (Soose et al. 2023) revealed that AChE inhibition was the principal factor distinguishing pesticide-exposed from non-exposed animals, even when considering motility variables (Sanchez-Hernandez, 2011; Lionetto et al. 2011; Legradi et al. 2018).

Recent studies have expanded our understanding of the role of these chemicals in motor activity. AChE is essential for regulating neuromuscular transmission and muscle contraction (Miyairi et al. 2024). According to (Rocha et al. 2024), AChE is crucial for modulating muscle peristalsis in earthworms across different soil types, highlighting its importance in locomotor behavior. Our study underscores the paramount importance of AChE activity inhibition as the primary indicator of significant differences between pesticide-exposed and non-exposed earthworms (Calisi et al. 2019). While motility variables provide valuable insights into earthworm behavior, our findings suggest that, when used on their own, they have limited capacity to fully quantify the effects of pesticide exposure (Djerdj et al. 2020). However, when integrated with AChE activity inhibition, motility variables enhance our understanding of earthworm responses to environmental stressors.

This highlights the complementary relationship between motility variables and AChE activity inhibition in providing a holistic assessment of pesticide-induced toxicity on earthworm populations. These results underscore the necessity of multifaceted approaches in ecological risk assessment and environmental monitoring endeavours (Calisi et al. 2019, Grassi et al. 2022).

Moreover, AChE is the enzyme involved in the hydrolysis of the neurotransmitter acetylcholine at the chemical synapse, thus terminating the nerve transmission signal and preventing the continuous depolarization or excitation of the postsynaptic cell (Lionetto et al. 2011; He et al. 2023). AChE activity is inhibited by organophosphorus compounds (Lionetto et al. 2011; Calisi et al. 2024) and, to a lesser extent, by methyl carbamates (Lionetto et al. 2011). This effect may be the main cause of the altered movement and related behavioural responses. Indeed, inhibition of AChE results in overstimulation of neuromuscular synapses with a state of prolonged contraction and consequent altered changes in locomotion and movement capacity; digging and burrow formation movement capacity; burrowing and burrow formation; alterations in tactile sensitivity and chemoreceptor activity; alterations in predation and escape activity; and aggression. It is therefore understood how motility tests can be useful in assessing soil contamination (Jouni et al. 2018, Calisi et al. 2019, Zidar et al. 2019, Ågerstrand et al. 2020).

The observed adverse effects indicate that carbamates can disrupt essential ecosystem services (nutrient cycling, organic matter decomposition, and soil structure maintenance) of non-target species, leading to broader ecological consequences. Therefore, our results underscore the need for further measures and research on carbamate and other types of pesticide impacts to safeguard soil ecosystems and their functions. In fact, as reported above, methiocarb is one of carbamates banned since 2020 in EU, but still in use in other part of the world (Henriques et al. 2020; Nartop et al. 2020; Tian et al. 2024) and still found nowadays in water sediments across Europe (Pizzini et al. 2024, Alves-Ferreira et al. 2024).

Moreover, ecotoxicological studies on non-target organisms are useful tools for assessing the potential toxicity of persistent contaminants, focusing on their bioavailable fraction. Locomotion (behavioural) tests could be considered an attractive tool, as they can reveal direct effects on the neuromuscular system (Sanchez-Hernandez, 2011; Jouni et al. 2018).

Further study with different contaminants at different concentrations will be necessary to achieve a complete standardization of locomotion tests in earthworms and to understand the mechanisms that induce behavioural alterations in locomotion levels in an ecotoxicological framework.

## Conclusions

This study shows the link between inhibition of acetylcholinesterase (AChE) activity and changes in locomotion in earthworms exposed to the carbamate pesticide methiocarb. In particular, altered trajectories, measured as changes in fractal dimension were assessed.

Motility tests can serve as excellent early indicators of high order level effects (organism, population, community, ecosystem) to comprehensively understand the ecological consequences of pesticide exposure.

## Data Availability

No datasets were generated or analysed during the current study.
